# Supplementary material for: NRG-HN003: Phase I and Expansion Cohort Study of Adjuvant Pembrolizumab, Cisplatin and Radiation Therapy in Pathologically High-Risk Head and Neck Cancer
Source: Cancers (Basel). 2021 Jun 9;13(12):2882. doi: 10.3390/cancers13122882 (PMC8230356; doi:10.3390/cancers13122882)
Supplement: Supplementary file 1 [file cancers-13-02882-s001.zip › cancers-1233259-supplementary.pdf]

Supplemental Table 1. Central Review of Radiation Therapy Compliance for Analyzable Patients in NRG-HN003 (n=34)

Review score: target volume contouring	
Per protocol	20 (58.8%)
Acceptable variation	10 (29.4%)
Unacceptable deviation	2 (5.9%)
Not evaluable	2 (5.9%)
Review score: organs at risk contouring	
Per protocol	27 (79.4%)
Acceptable variation	4 (11.8%)
Unacceptable deviation	1 (2.9%)
Not evaluable	2 (5.9%)
Review score: target volume and organs at risk contouring	
Per protocol	20 (58.8%)
Acceptable variation	10 (29.4%)
Unacceptable deviation	2 (5.9%)
Not evaluable	2 (5.9%)
Review score: target volume dose volume analysis	
Per protocol	23 (67.6%)
Acceptable variation	9 (26.5%)
Not evaluable	2 (5.9%)
Review score: organs at risk dose volume analysis	
Per protocol	29 (85.3%)
Acceptable variation	3 (8.8%)
Not evaluable	2 (5.9%)
Review score: total dose	
Per protocol	31 (91.2%)
Unacceptable deviation	1 (2.9%)
Not evaluable	2 (5.9%)

Supplemental Table 1. Central Review of Radiation Therapy Compliance for Analyzable Patients in NRG-HN003 (n=34)

Review score: fractionation	
Per protocol	31 (91.2%)
Unacceptable deviation	1 (2.9%)
Not evaluable	2 (5.9%)
Review score: elapsed days	
Per protocol	29 (85.3%)
Acceptable variation	1 (2.9%)
Unacceptable deviation	2 (5.9%)
Not evaluable	2 (5.9%)
Review score: overall	
Per protocol	18 (52.9%)
Acceptable variation	11 (32.4%)
Unacceptable deviation	2 (5.9%)
Incomplete radiation therapy, progression	1 (2.9%)
No radiation therapy given	2 (5.9%)

Supplemental Table 2. Central Review of Cisplatin Compliance for Analyzable Patients in NRG-HN003 (n=34)

Review score: overall	
Per protocol	27 (79.4%)
Acceptable variation	5 (14.7%)
Not evaluable	2 (5.9%)
Review score: dose	
85-115%	16 (47.1%)
< 85% due to protocol-specified reasons	13 (38.2%)
70 - < 85% due to non-protocol-specified reasons	3 (8.8%)
Not evaluable	2 (5.9%)
Review score: delays	
No delays	23 (67.6%)
≤ 1 week	9 (26.5%)
Not evaluable	2 (5.9%)

Supplemental Table 3. Central Review of Pembrolizumab Compliance for Analyzable Patients in NRG-HN003 (n=34)

Review score: concurrent overall	
Per protocol	31 (91.2%)
Acceptable variation	1 (2.9%)
Unacceptable deviation	2 (5.9%)
Review score: concurrent dose	
85-115%	29 (85.3%)
< 85% due to protocol-specified reasons	3 (8.8%)
< 70% due to non-protocol-specified reasons	2 (5.9%)
Review score: concurrent delays	
No delays	26 (76.5%)
≤ 1 week	8 (23.5%)
Review score: maintenance overall	
Per protocol	30 (88.2%)
Acceptable variation	2 (5.9%)
Not evaluable	2 (5.9%)
Review score: maintenance dose	
85-115%	19 (55.9%)
< 85% due to protocol-specified reasons	12 (35.3%)
70 - < 85% due to non-protocol-specified reasons	1 (2.9%)
< 70% due to non-protocol-specified reasons	2 (5.9%)
Review score: maintenance delays	
No delays	20 (58.8%)
≤ 1 week	11 (32.4%)
> 1 week due to protocol-specified-reasons	2 (5.9%)
Not evaluable	1 (2.9%)
